# Non-coding RNAs in pluripotency and neural differentiation of human pluripotent stem cells

**DOI:** 10.3389/fgene.2014.00132

**Published:** 2014-05-14

**Authors:** Dunja Lukovic, Victoria Moreno-Manzano, Martin Klabusay, Miodrag Stojkovic, Shomi S. Bhattacharya, Slaven Erceg

**Affiliations:** ^1^Retina Group, Cell therapy and Regenerative Medicine, Centro Andaluz de Biología Molecular y Medicina RegenerativaSevilla, Spain; ^2^Neuronal and Tissue Regeneration Lab, Centro de Investigación Príncipe FelipeValencia, Spain; ^3^Integrated Center of Cellular Therapy and Regenerative Medicine – International Clinical Research Center, St. Anne’s University Hospital BrnoBrno, Czech Republic; ^4^Spebo MedicalLeskovac, Serbia; ^5^Human Genetics Department, Faculty of Medical Sciences, University of KragujevacKragujevac, Serbia

**Keywords:** pluripotent stem cells, pluripotency, non-coding RNA, differentiation, human embryonic stem cells

## Abstract

Several studies have demonstrated the important role of non-coding RNAs as regulators of posttranscriptional processes, including stem cells self-renewal and neural differentiation. Human embryonic stem cells (hESCs) and induced pluripotent stem cells (ihPSCs) show enormous potential in regenerative medicine due to their capacity to differentiate to virtually any type of cells of human body. Deciphering the role of non-coding RNAs in pluripotency, self-renewal and neural differentiation will reveal new molecular mechanisms involved in induction and maintenances of pluripotent state as well as triggering these cells toward clinically relevant cells for transplantation. In this brief review we will summarize recently published studies which reveal the role of non-coding RNAs in pluripotency and neural differentiation of hESCs and ihPSC.

## INTRODUCTION

Personalized medicine is expected to benefit from the combination of genomic information with the high throughput studies including transcriptomic, proteomic and metabolomic profiling. Measuring gene expression in individual cells is crucial for understanding the gene regulatory network. In order to decipher the genetic regulatory network in cells significant efforts have been made over the years to develop technology platforms for transcriptome characterization such as DNA microarray hybridization, serial analysis of gene expression (SAGE; Velculescu et al., 1995) or next-generation RNA sequencing often called RNA-seq (Mortazavi et al., 2008).

The latest techniques which involve bioinformatic expertise made a revolution in transcriptome analysis enabling not only the identification of cDNA and gene isoforms but discovery of long non-coding RNA (large intergenic non-coding RNA, lincRNA; >200 nucleotides in length) and short non-coding RNA (sncRNA, <200 nucleotides in length). Non-coding RNAs include transfer RNA (tRNA), ribosomal RNA (rRNA), small nuclear and small nucleolar RNA, microRNA (miRNA), and small interfering RNA (siRNA), which do not encode any proteins. Several of these non-coding RNA species, like miRNA or SiRNAs, are of particular interest to transcriptomic and particularly in stem cell research due to their role in post-transcriptional regulation of numerous biological processes (Morozova and Marra, 2008; Roukos, 2010). During the last several years many studies were published in order to determine the function of these non-coding transcripts including novel miRNA (Hafner et al., 2008) that exhibit different cell-type and tissue specificity (Guttman and Rinn, 2012). Although the functions of the majority of newly discovered non-coding RNAs are still unknown, some were found to play important roles in the regulation of stem cells. Recent studies concentrate on miRNAs (Wilson et al., 2009; Kim et al., 2011; Lipchina et al., 2011). In the context of stem cell biology, of particular interest is the role of these RNAs in expression of renewal genes in human embryonic stem cells (hESCs) or in regulation of induced pluripotency (Li et al., 2011). In this review, we focus on recent discoveries of non-coding RNA roles in human pluripotent stem cell biology and differentiation.

## HUMAN EMBRYONIC STEM CELLS AND INDUCED PLURIPOTENT STEM CELLS

Human pluripotent stem cells encompassing hESCs and induced pluripotent stem cells (ihPSCs) show great potential for regenerative biology providing the unique human *in vitro* platforms for studying diseases, basic cell biology and development.

Human embryonic stem cells can be derived from inner mass from human blastocyst maintaining unique capacity for unlimited self-renewal through long-term maintenance using laboratory culture conditions (Thomson et al., 1998). Since the generation of the first hESCs line in 1998 (Thomson et al., 1998), research in this area has progressed at a rapid pace, developing efficient protocols globally for differentiation of these cells to clinically relevant cell types (Erceg et al., 2008, 2009, 2010, 2012). hESCs represent a useful model for studying early human embryology and cell differentiation and have limited capacity for disease modeling in human cells (Biancotti et al., 2010). hESCs bear the advantage over any other stem cells in that they are pluripotent, providing an unlimited starting cell source for differentiation to any type of tissue of the human body. The perspective of clinical use of these cells and their derivates is huge. The hESCs-based therapy is increasingly recognized as a promising strategy for degenerative disorders entering already in clinic to treat spinal cord injury or recently published encouraging results in human clinical trial investigating their use in age-related macular degeneration (Schwartz et al., 2012). The main disadvantage of use of hESCs in regenerative medicine is the fact that derivation of hESCs requires the destruction of human embryos which generates the ethical concerns.

Besides the abundance and efficient differentiation without traces of pluripotency, the main requisite for personalized regenerative medicine is to derive disease cells that genetically match the patient. Although the technique of somatic cell nuclear transfer (SCNT) and successive derivation of hESCs (Tachibana et al., 2013) could be a promising approach in the future to create patient specific cells, major technical and ethical obstacles related with this technique are present.

The discovery of human ihPSCs originally generated by ectopic expression of four transcription factors Oct4, Sox2, Klf4, and cMyc (Takahashi et al., 2007) in human fibroblast cells presents a novel tool to obtain disease cells. This Nobel Prize winner technology was substantially improved by introducing non-integrative transgene expression (Jin et al., 2012) and targeting different somatic tissues. Patient-specific ihPSCs derived from somatic cells are devoid of immnunological and ethical concerns, allow the generation of disease-specific stem cells providing a platform to study molecular mechanisms of genetic diseases. The ihPSCs show morphological, transcriptional, epigenetic, and phenotypic similarity to hESCs and can differentiate toward any cell of human body. Until now a number of studies has shown that ihPSCs can be successively generated from patients carrying different diseases and be a faithful platform for disease modeling *in vitro* (Gunaseeli et al., 2010; Hargus et al., 2010; Jin et al., 2011, 2012; Pedrosa et al., 2011; Kumano et al., 2012; Oh et al., 2012; Sun et al., 2012; Cocks et al., 2013; Gross et al., 2013; Tubsuwan et al., 2013).

Pluripotent stem cells possess two major characteristics: self-renewal and differentiation into other cell types. The investigators put the major effort in development of new protocols and moving these cells to clinics but it is crucial to understand these two main characteristics in order to enter deeply in basic biology of these cells. For example it is still to be elucidated reprogramming mechanisms in target cells and why only small population of cells becomes fully reprogrammed. In order to decipher molecular mechanisms of reprogramming the role of RNA and related global gene expression changes is of particular interest in order to increase reproducibility and efficiency of reprogramming processes. Reproducible generation of specific cellular type without traces of ihPSCs is one of the crucial issues in order to prevent teratoma generation in host. Improvements of the differentiation protocols are required as a basis for further cost-efficient industrial processes of large-scale for future application in clinics. To reach this also extensive characterization of differentiated cell has to be performed and subsequently compared with undifferentiated counterparts. Comparative transcriptome analyses using microarray also indicate that hESCs and hiPSCs have similar, highly alike gene expression patterns. Gene expression pattern of ihPSCs is separate from the originating somatic cells with possibility of retaining some transcriptional differences or an epigenetic memory of the starting cells (Plath and Lowry, 2011). Transcriptome characterization would undoubtedly provide insights into the genetic regulatory networks involved in maintaining pluripotency and directing differentiation. In order to define molecularly the various phases of the reprogramming process, as well as full pluripotent stem cells state global gene expression and proteomic patterns of clonal cell populations or enriched populations need to be performed in different stages after inicial reprogramming induction.

## PLURIPOTENCY

Generally, a definition of pluripotency is related to ability of cell to give rise three germ layers: endoderm, ectoderm, and mesoderm and their derivates. This ability has only a small number of cells such as hESCs and ihPSCs and their maintenance involves core transcription factors: Oct4, Sox2, and Nanog (Boyer et al., 2005; Kim et al., 2009). A spectrum of different miRNA was detected in embryonic stem cell as pluripotency-specific markers which expression was downregulated during the induction of differentiation (**Table 1**; Wilson et al., 2009; Lee et al., 2010). A family of miRNA that includes AAGUGC seed sequence is of particular interest in pluripotent stem cells for its high expression in hESCs and ihPSC. The most abundant miRNA transcript in hESCs is *mir-302 *which encodes for miR-302a/b/c/d and mir-367 (Suh et al., 2004) and is under the control of Oct4, Sox2, and Nanog. This miRNA is involved in maintenance of pluripotency, self-renewal, regulation of cell cycle, and fate specification during differentiation of hESCs (Suh et al., 2004; Landgraf et al., 2007; Bar et al., 2008; Lipchina et al., 2011) probably inhibiting neural differentiation by modulation of BMP signaling targeting its inhibitors: TOB2, DAZAP2, and SLAIN1 (Lipchina et al., 2011). Rosa and Brivanlou (2011) have shown that Oct4 and miR-302 inhibit NR2F2, which in turn inhibits Oct4. The expression of gene *NR2F2* is increased during differentiation when the expression of *OCT4* gene and miR-302 declines (Rosa and Brivanlou, 2011). This study showed important biological function of mir-302 and NR2F2 in human early development and cell fate determination. It seems that other miRNAs such as miR-145 has the opposite role in maintenance of pluripotency (Xu et al., 2009). The expression of this miRNA is low in undifferentiated hESCs but its increased expression is related to inhibition of hESCs self-renewal and induction of lineage-restricted differentiation (Xu et al., 2009).

**Table 1:** Different roles of non-coding RNA in pluripotency and neural differentiation.

Type of cells	Processes involved	Non-coding RNA	Reference
hESC	Pluripotency, self-renewal, cell cycle and fate specification	miR-302	Suh et al. (2004), Bar et al. (2008), Lipchina et al. (2011)
hESC	Inhibition of pluripotency	miR-145	Xu et al. (2009)
iPSC	Pluripotency	miR-17, miR-106b, and miR-106a	Li et al. (2011)
Fibroblasts to iPSC	Reprogramming	miR-302, miR-372	Anokye-Danso et al. (2011),^2012^, Subramanyam et al. (2011)
Fibroblasts to iPSC	Reprogramming	Combination of miR-302, miR-200c, and miR-369	Miyoshi et al. (2011)
iPSC	Reprogramming	LincRNAs	Loewer et al. (2010)
hESC	Neural differentiation	LincRNAs	Ng et al. (2012)
iPS-derived neural progenitors	Neural differentiation	LincRNAs	Lin et al. (2011)
hESC	Differentiation to neuroectoderm	miR-200, miR-96	Du et al. (2013)
hESC-derived neural stem cells	Suppression of selfrenewal, neural differentiation	miR-124, miR-125b and miR-9/9	Roese-Koerner et al. (2013)
hESC	Neural differentiation	miR7	Liu et al. (2012)
hESC	Neural differentiation	miR125	Boissart et al. (2012)

*hESC, human embryonic stem cells; iPSC, induced pluripotent stem cells.*

Elucidation of the precise molecular and cellular mechanisms which convert human fibroblasts or other somatic cells to ihPSCs was the main challenge among the investigators during the last years. Reprogramming somatic cells into pluripotent cellular identity requires tightly regulated and coordinated changes in expression of many genes. Understanding the genetic network involved in cellular reprogramming is crucial to elucidate pluripotency in order to increase the reprogramming efficiency and cell renewal. These mechanisms will reveal why only small population of somatic cells undergo full reprogramming. Different gene expression patterns and post-transcriptional events, including mRNA decay, between pluripotent and differentiated cells could reveal the reprogramming mechanisms of the fibroblasts into ihPSCs. The study of Buganim et al. (2012) showed that reprogramming involves stochastic gene expression in early phase followed by a late hierarchical phase with activation of *SOX2* gene, which then triggers a stepwise gene activation that allows the cells to enter the pluripotent state. SOX2 represents a group of pluripotency initiating factors (PIFs) indispensable for endogenous activation of *OCT4*, *SOX2,* and *NANOG* (Boyer et al., 2005) which further maintain the ihPSCs state. Some of these genes maintain pluripotency by blocking the gene machinery involved in differentiation.

In the study of Li et al. (2011) was observed that three miRNA clusters: miR-17, miR-106b, and miR-106a were significantly upregulated that interfere with iRNA machinery directly connected with important reprogramming pathways: TGF-β signaling and cell cycle. These results suggest that transcription factors that modulate miRNA decay could have crucial role in reprogramming differentiated cells or in maintaining pluripotency, but future studies have to be performed to confirm whether these factors can be efficient target to induce or maintain the pluripotency or trigger the differentiation.

Several miRNA, especially miR-302 and miR-372 have been directly involved in enhancing of HFF reprogramming (Subramanyam et al., 2011) revealing the possibility to directly target these miRNAs to reprogram the HFF without Yamanaka factors. The recent study of Morrisey and colleague (Anokye-Danso et al., 2011, 2012), confirmed that reprogramming can be achieved by using miRNAs without protein-coding factors. Another study confirmed that fully pluripotent stem cells can be obtained by introducing other miRNA such as combination of miR-302, miR-200c, and miR-369 (Miyoshi et al., 2011). Different studies speculated about the mechanisms and signaling pathways by which these miRNAs exert their reprogramming function such as regulation of different genes involved in cell cycle, epithelial-mesenchymal transition, epigenetic regulation and vesicular transport (Subramanyam et al., 2011).

On the other hand, the abundance of lincRNAs in mammalian transcriptome reveals their role as key regulators of biological processes. These RNA transcripts have little or no protein coding potential but some studies point out their possible participation in pluripotency, differentiation and self-renewal (Guttman et al., 2009, 2011; Sheik Mohamed et al., 2010; Guttman and Rinn, 2012). Several studies have recently discovered a novel class of lincRNAs possible involved in reprogramming processes, pluripotency and lineage commitment (Boyer et al., 2006; Lee et al., 2006; Loewer et al., 2010).

Some of these lincRNAs act directly as regulators of reprogramming (RoR) called lincRNA-RoR (Loewer et al., 2010). Overexpression of these RNAs significantly enhances the reprogramming efficiency and their downregulation decreases the generation of ihPSC colonies possibly by mechanism of negative regulation of p53 (Zhang et al., 2013).

These studies indicate that non-coding RNAs, especially miRNAs have the potential to be used as small-molecule therapeutics to promote more efficient reprogramming or to induce the pluripotent stem cells toward other cell lineages.

## DIFFERENTIATION

In the context of regenerative medicine it is crucial to develop protocols for efficient and reproducible differentiation of pluripotent stem cells toward homogeneous population of desired cells without traces of pluripotency. Since the generation of the first hESCs line (Thomson et al., 1998) and derivation of ihPSC (Takahashi et al., 2007), research in this area has progressed at a rapid pace, developing efficient protocols globally for differentiation of these cells to clinically relevant cell types. As already mentioned, hESCs and ihPSCs bear the advantage over any other stem cells in that they are pluripotent, providing an unlimited starting cell source for differentiation to any type of tissue of the human body. Understanding the regulatory mechanisms which orchestrate the hESCs and ihPSCs during differentiation is of enormous importance because coordinated changes in gene expression during the differentiation of hESC and ihPSC are crucial for lineage specification. Beside the gene expression changes in coding RNA it is a clear to investigate whether non-coding RNA play important role in early differentiation of pluripotent stem cells. Although recent studies have shown that ihPSCs lines exert better differentiation capacity when compared with hESCs (Hu et al., 2010) direct comparison of differentiated cells versus undifferentiated counterparts is crucial in order to find signaling mechanisms involved in differentiation. In the recent study Gifford et al. (2013) performed comprehensive transcriptional profiling of cell populations generated by directed differentiation of hESCs.

To reveal whether lincRNAs play important role in hESCs and neural differentiation Stanton and colleague (Ng et al., 2012), employed a highly efficient protocol for neural differentiation of hESCs based on stromal-derived induction activity (SDIA) using co-culture of hESCs with PA6 mouse stromal cells. This procedure, used by many groups, was designed to generate homogeneous population of neural progenitor cells and further dopaminergic neurons (Kawasaki et al., 2000, 2002; Zeng et al., 2004). About 36 lincRNAs were identified which were associated with pluripotency making the complex with SOX2, and SUZ12, well known genes involved in pluripotency. Association of newly discovered lincRNAs with MIR-125B and LET7A reveal important role of these lincRNA in neurogenesis and neural differentiation. These results demonstrate that lincRNAs represents indispensable components in regulation of biological processes such as neural differentiation and pluripotency.

In order to clarify the contribution of lincRNA in developmental and neurological disorders, Lin et al. (2011) were performed Genome-wide analysis using next-generation sequencing (RNA-Seq) of neural progenitors derived from ihPSCs. They found that early differentiated cells underwent dramatic quantitative changes in gene expression especially lincRNAs. The authors associated many lincRNAs with *HOX* gene (*HOXA* and *HOXB*), genes involved in early patterning of anterior posterior axis during the neural development. These results coincided with results obtained with neural progenitors derived from hESCs as an additional prove that these two sources of pluripotent stem cells has similar neuronal differentiation potential (Wu et al., 2010). The author’s general aim in this article is to associate the obtained results with some neuropsychiatric disorders in order to establish faithful lincRNA markers. The RNA-Seq findings highlighted possible non-coding RNA variants as feasible candidates which mutations are involved in many neuropsychiatric disorders mostly schizophrenia, bipolar disorders and autism spectrum disorders. These transcription factors and chromatin modifiers candidate are: *POU3F2*,* MYTIL*,* RFX4*,* ZNF804A*,* SMARCA2, *and* NPAS3. *These changes in the transcriptome profiles and the role of lincRNA during early human neural differentiation using pluripotent stem cells reveals important use of ihPSC technology in studying human disease as a unique human assay of human neurogenesis. Integration the novel transcripts in more global systems of analysis is must in order to elucidate their abnormally regulation in a subgroup of patients.

Comparing the miRNA profiles of neuroectodermal cells to epidermal cells both derived from hESC, Zhang and colleague (Du et al., 2013) identified the downreglation of two miRNA families in neuroectodermal differentiated cells, miR-200 and miR-96. Investigating the function of these miRNA it was discovered that miR-200 regulates the level of zinc-finger E-box-binding homeobox (ZEB), transcription factor family involved in inhibition of expression of BMP and its downstream genes, thus promoting neural differentiation (Postigo et al., 2003), while miR-96 regulates PAX6 (paired box 6), well known transcription factor characteristic for neuroectoderm. The authors also find that upregulation of these miRNA suppresses differentiation of hESCs toward neural lineage (Du et al., 2013). Recent article examined the role of the neural-associated miR-124, miR-125b, and miR-9/9 in human neural stem cells derived from human pluripotent stem cells (Roese-Koerner et al., 2013) and showed that overexpression of these miRNA suppress self-renewal and induce further differentiation into neurons. Providing additional evidence of involvement of other miRNA such as miR7 (Liu et al., 2012) and miR125 (Boissart et al., 2012) in neural differentiation of hESCs, these studies showed that neural stem cells derived from pluripotent stem cells could be a faithful model for investigation of role of miRNA in modulating of stemness and neuronal differentiation capacity of these cells.

## CONCLUSION

Studying of non-coding RNA in modeling exhaustive networks of gene interactions as an ultimate application of systems biology in systems biomedicine, could substantially contribute to understanding and modulation of developmental and differentiation processes in humans. Although the expression of newly correlated non-coding RNA is strongly associated to pluripotency and neural differentiation their possible role in different neurodegenerative disorders is still to be elucidated. These studies undoubtedly contribute to better understanding of the biological processes during pluripotency and neural differentiation and reveal the important interplay between multiple pluripotency transcription factors and non-coding RNAs especially miRNAs. However, the understanding of the impact of miRNA-based regulation in human neural development is still at its dawn. The future studies will confirm the potential of controlling differentiation and pluripotency of human pluripotent stem cells by modulating the expression of selected non-coding RNAs and integrate them into models that reveal the global behavior of the biological process in biomedicine and neural diseases in order to ultimately improve patients’ quality of life.

## Conflict of Interest Statement

The authors declare that the research was conducted in the absence of any commercial or financial relationships that could be construed as a potential conflict of interest.
